# Female Labor Force Status and Couple's Marital Satisfaction: A Chinese Analysis

**DOI:** 10.3389/fpsyg.2021.691460

**Published:** 2021-07-23

**Authors:** Xiao Yu, Shu Liu

**Affiliations:** Northeast Asian Research Center, Jilin University, Changchun, China

**Keywords:** China, marital satisfaction, female labor participation, work scheduling, gender role attitudes

## Abstract

While China has decreasing female labor participation and increasing marital instability, compared to the rest of the world, its female labor participation rate is higher on average. The effect of female labor force status on couples' marital satisfaction, as one of the main factors for evaluating marital quality, has been separately discussed, including extensive margins considering whether women are in the labor market and intensive margins on women working hours per week. This study analyzed data from the 2014 China Family Panel Studies (CFPS) using a binary logit model and a stability test. Results showed that the work hours, rather than the occupational status, of women affect marital satisfaction. In addition, regardless of the gender role attitudes held by the couple, marital satisfaction increases when women are in the labor market. This study has retroactively reviewed the effects of women working outside the home on marital quality. The dual roles of Chinese women, as both employee and homemaker, have been socially accepted. However, the requirements of maintaining multiple roles often contradict and present conflicts among the roles, time, and pressure, in the long run, giving rise to marital dissatisfaction.

## Introduction

**Figure 1:**
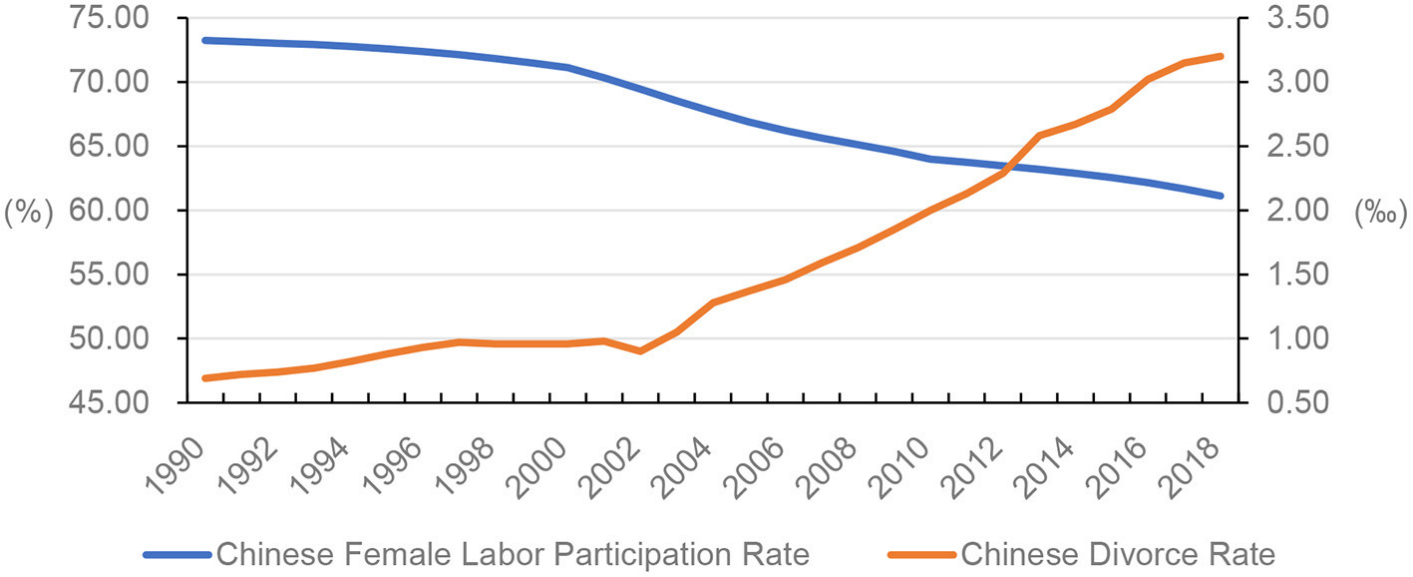
Chinese female labor participation rate (%) and Chinese crude divorce rate (%0), 1990–2018. Female labor force participation rate is the proportion of the female population aged 15 years and older that is economically active and modeled based on ILO estimate. Chinese crude divorce rate is the number of divorces per 1,000 people in China. *Sources:* World Bank. (2020) and China Social Statistic Yearbook of 2019. (2020).

Most developed countries experience an increase in female labor participation and marital instability simultaneously (Greenstein, 1990; Rogers, 1999; Sayer and Bianchi, 2000). However, in China, the trend features increased marital instability and decreased female labor participation (see Figure 1).

**Figure 2:**
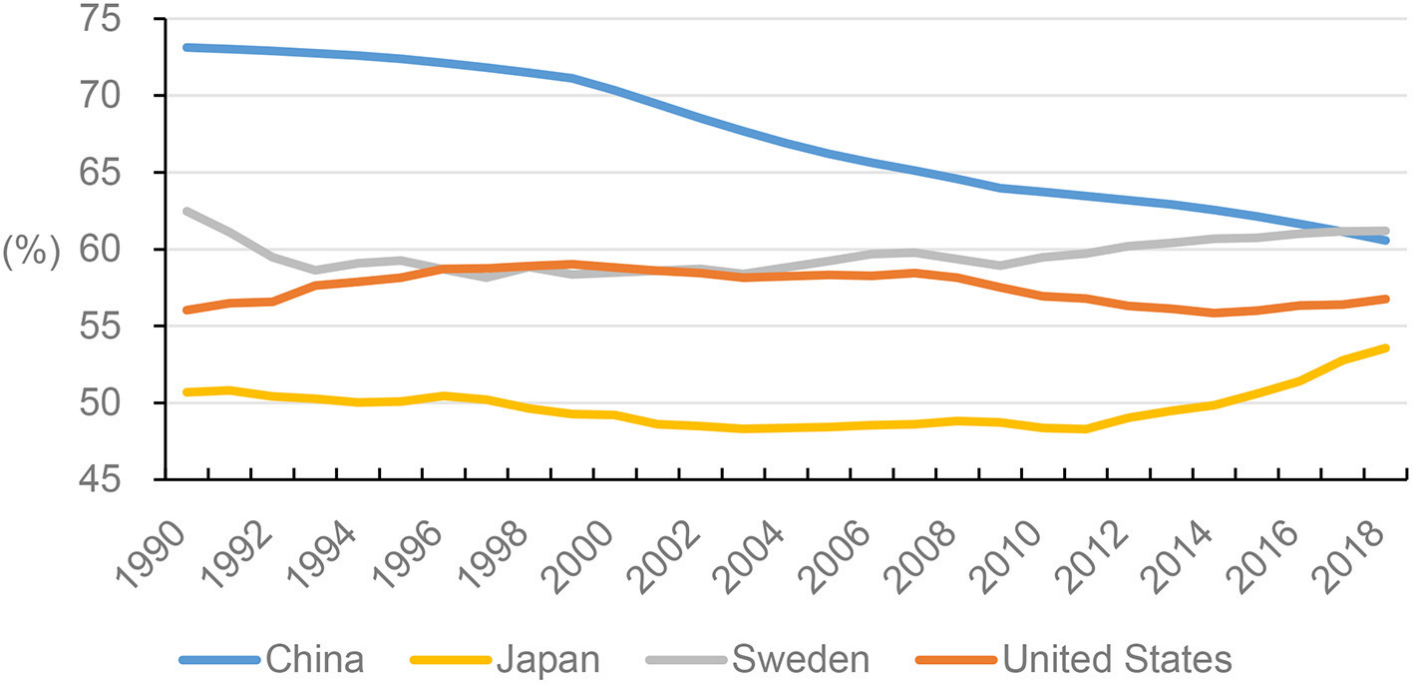
Female labor participation rate (%) among China, the US, Sweden, and Japan, 1990–2018. Female labor force participation rate is the proportion of the female population aged 15 years and older that is economically active and modeled based on ILO estimate. *Sources:* World Bank. (2020).

Although China has a higher female labor participation rate compared to other countries, the female labor participation rate in China has decreased dramatically, from 73% in 1990 to 61% in 2018 (see Figure 2). Gender equality policies from plan-oriented economies contributed to the increased labor participation rate between 1949 and 1978 in China, reaching about 90%. However, during that era, overworked women were a common phenomenon (Pan, 2002; Li and Li, 2008). With the transfer to a market-oriented economy after 1979, macro-level factors of the market system, economy development, and micro-level factors related to family needs have mutually contributed to the decrease in female labor participation. In a market-oriented economy, the market is the chief driver of labor allocation, not the government. Females were able to make independent decisions regarding whether to enter the labor market. Thus, part of the decline in female labor participation could be attributed to individual decisions (Pan, 2002). Organizations transformed into rational employers to balance costs and benefits. Female and male employees started to compete for the same positions. Due to medical liabilities unique to the female gender and gender discrimination toward assumed insufficient female productivity, female employees lost their competitive advantage, which partly explained the decline in female labor participation (Pan, 2002; Li and Li, 2008; Wu, 2010). With economic development, however, the rise in individual income levels increased the demand for leisure time; therefore, some females left the labor market. Through this process, domestic labor division was strengthened (Ding, 2008). Since China's economic reform, government-supported caretaking has been gradually terminated, shifting childrearing, and eldercare back to households (Ji, 2015; Qi and Dong, 2018). Therefore, the traditional expectation from women to care for children and the elderly has also contributed to low female labor market participation (Ma et al., 2011; Zhu, 2019). Therefore, gender inequality in the country is reinforced by political and economic decisions, and Chinese women who wish to work undertake two opposing roles—being full-time employees and homemakers—which reveals a discrepancy between egalitarian household economic relationships and complementarian household labor divisions, ultimately increasing tension within families (Robinson, 1985; Zhu, 2019).

Since the 1980s, Chinese attitudes toward marriage and family have transitioned from a strict traditional hierarchical system to a relatively relaxed modern one, focusing on individualism, and an egalitarian relationship between an individual and the family members (Ma et al., 2011). This transition consequently has altered family formation, structure, size, and the relationships between husbands and wives, and between parents and children (Xu and Ocker, 2013; Ma et al., 2018; Liu, 2019). A new marriage law, enacted in 1981, featured a simplified divorce procedure that shifted control of marital decisions from extended families to individuals, who became empowered to independently determine whether to begin or end a marriage, especially enabling development for women (Pimentel, 2000; Xu and Ocker, 2013; Ma et al., 2018). Thus, social acceptance of divorce is increasing, and people can pursue divorce without limitations. Indeed, a vast majority of divorce proceedings have been initiated by women (Platte, 1988; Xu, 2012). Thus, the divorce rate increased from 0.18‰ in 1978 to 3.20‰ in 2018 (see Figure 1).

Most studies have concentrated on marital effects on female labor participation. Theories of domestic labor division and work-to-family conflicts explain marital effects on gender differences in labor participation, where female employees are more likely than male employees to postpone work tasks due to family issues (Ogawa and Ermisch, 1994; Yu and Lee, 2013; Cai, 2019; Jiang and Dai, 2019; Wu, 2019). However, few studies have explained the retroactive effects of female labor participation on marital issues. Furthermore, female labor participation and marital stability have a bilateral relationship. On the one hand, there is no clear causal relationship between female labor participation and marital instability. Based on gender role differences, the loss of economic power exchange between the genders when women are employed and the independence of working women have contributed to the positive causal relationship between a rise in female labor participation and increasing marital instability (Parsons, 1949; Ross and Sawhill, 1975; Booth et al., 1984; Becker, 1991). However, Greenstein (1990), Oppenheimer (1997), Rogers (1999), and Sayer and Bianchi (2000) found little empirical support for the positive causal relationship between female labor participation and marital instability. On the other hand, there does exist a clear positive causal relationship between marital instability and female labor participation. Increase in marital instability contributes to the rise of female labor participation, especially for women with high divorce risk and who are not employed (Johnson and Skinner, 1986; Greenstein, 1990; Ogawa and Ermisch, 1994; Rogers, 1999). Xu and Ocker (2013) argue that although the topic of family life has not been extensively researched in China, marital stability is essential to a family-centered society, in that it is intricately connected to fertility and aging issues. Ma et al. (2018) further posed a question that required research regarding how women's labor participation development affects marital instability in China.

Thus, whether female labor participation is a reason for marital dissolution is unclear. The argument positing a positive relationship between marital quality and marital stability is empirically supported by Booth et al. (1985) and Xu (2010). Xu (2012) further supports that marital quality not only directly and positively affects marital stability but is also an intermediary to other factors, ultimately affecting marital stability. Furthermore, marital satisfaction, as a crucial index of marital quality, has positively influenced marital stability and contributes to spiritual and physical health (Wilcox and Nock, 2006). Indeed, marital satisfaction itself also has direct and indirect positive effects on marital stability, in that a satisfied couple contributes to a stable marriage. Marital satisfaction itself is a useful predictor for marital stability; also, it is a mediator for other factors affecting marital stability (Hicks and Platt, 1970; Udry, 1981; White and Booth, 1991; Sanchez and Gager, 2004).

Therefore, in this study, marital satisfaction was chosen to measure couples' marital quality. Subsequently, both the extensive and intensive effects of female labor participation on marital satisfaction for women and their spouses were investigated separately, including extensive inquiries regarding the presence of women in the labor market and intensive inquiries regarding the quantity of time devoted to jobs. A binary model of marital satisfaction was established, and the China Family Panel Studies (CFPS) of 2014 were reviewed to discuss significant issues.

## Hypotheses

### Effects of Women's Labor Participation on Marital Satisfaction Based on Gender

Research on marital quality and female employment is centered on whether and how women's employment affects marital quality, including comparisons between paid and unpaid labor, and between full-time and part-time employment (Ye and Xu, 2000). In addition, there are gender differences in marital quality evaluation, where Bernard (1972) argues that women experience lower marriage satisfaction than men. However, Jackson et al. (2014) find little evidence to support gender differences in marital satisfaction. Additionally, wives are affected by marital characteristics and perceptions of unfairness, while husbands are affected by their employment (Vannoy and Philliber, 1992; Blair, 1998; Wilcox and Nock, 2006; Jackson et al., 2014). Nock (2001) further argues that men are more significantly advantaged by their marriages than women because men have benefited from the status of being married, regardless of marriage quality. Conversely, women are more likely to describe marital advantages based on quality experiences.

Thus, this study developed Hypothesis 1: Women's labor participation has different effects on marital satisfaction based on gender. Wives' labor participation may decrease husbands' marital satisfaction. But wives' labor participation may not affect their own marital satisfaction.

### Effects of Gender Roles on Marital Satisfaction

Gender role is a crucial factor in marital stability, which is supported by gender role functional theory by Parsons (1949) and gender role and competition theory by Becker (1991). Gender roles also affect marital satisfaction, fertility decisions, domestic labor division, and labor participation decisions (Feng and Xiao, 2014). Furthermore, individuals are more likely to encounter marital problems when their counterparts' gender role attitudes exceed their own expectations (Vannoy and Philliber, 1992; Li et al., 2017). Vannoy and Philliber (1992) emphasized that gender roles, not female labor participation, affect marital quality. Traditional gender role attitudes toward women negatively impact human capital acquisition, educational return, and labor supply (Kosteas, 2013). However, in more gender-equal societies, women and their husbands are more significantly incentivized to support female labor participation (Yu and Lee, 2013). Over the past 20 years, Chinese women have become more egalitarian than men, while men have remained more conservative in every age cohort (Liu and Tong, 2014; Yang, 2014; Sun, 2018). Indeed, Liu and Tong (2014) conclude that women who are more egalitarian toward gender roles are typically classified in higher socioeconomic statuses, make financial contributions to their families, have equivalent or higher occupational statuses, and exert familial assertiveness with their counterparts.

Thus, this study developed Hypothesis 2: Under traditional gender roles, wives' labor participation decreases both wives' and husbands' marital satisfaction.

### Effects of Domestic-Labor Division on Marital Satisfaction

Despite changes in the workforce, the housekeeping role of women persists, and the gender gap among the share of housework remains statistically unchanged. The average time devoted to performing household chores for women per day is 2.1 h, which is 2.4 times higher than that for men (0.75 h). Women also engage in 53 min of childrearing, compared to 17 min for men (National Bureau of Statistics of China., 2019). Yang (2014) concludes that gender roles in China have become more egalitarian, but attitudes around domestic labor division tend to be conservative. Most employed Chinese women work full-time jobs but are still responsible for unpaid care work; therefore, women have double or triple roles as dual-earners, housekeepers, and oftentimes caregivers (Yang, 2014; Qi and Dong, 2018). Thus, with the burden of housework, women encounter psychological and emotional risks, which indirectly affect marital quality. Husbands' reluctance to share household responsibilities will increase marital tension, especially for women who believe in egalitarian gender roles (Robinson, 1985; Blair, 1998; Wilcox and Nock, 2006; Jackson et al., 2014). Additionally, wives who perform less housework will decrease their husbands' marital satisfaction (Zhu and Qiao, 2015).

Thus, this study developed Hypothesis 3: Husbands who participate in domestic work may increase their wives' marital satisfaction but decrease their own marital satisfaction.

### Effects of Women's Work Hours on Marital Satisfaction

Although women's dependencies on men have decreased with an increase in their own economic statuses, the bargaining power of resource exchanges, and traditional gender distribution patterns still contribute to a couple's time allocation between market and domestic labors (Cui, 2017). While overtime work is not socially acceptable in China, Qi and Dong (2018) highlight that it is common in China, especially in manufacturing and commercial service sectors. Additionally, women who work in rural regions work more hours than their urban counterparts. Wives' work hours have substantial effects on marital quality, negatively impacting marital interaction and positively contributing to family conflict (Blair, 1998; Wu et al., 2015). Wives' high workloads may increase their psychological pressure when they or their counterparts hold traditional gender attitudes (Steiner et al., 2019).

Thus, this study developed Hypothesis 4: Wives who work extended hours decrease their husbands' marital satisfaction.

### Effects of Women's Income on Marital Satisfaction

Blair (1998) argues that marital quality is measured by factors such as hours spent in the workplace, income, and work scheduling. Becker (1991) emphasizes that increasing public wages will affect female labor participation and marital quality by attracting women to the labor market and increasing marital risks. Ogawa and Ermisch (1994) further argue that women with low earning husbands experience financial strain and increase the likelihood of divorce. Women prefer to marry men with better socioeconomic conditions. Gender income differences perpetuate a conservative pattern of domestic labor division, given that women, on average, earn lower wages than men. However, if women have a higher income and occupational status than their husbands, and husbands are reluctant to accept this development, married couples are likely to experience a decrease in marital satisfaction and increase in the risk of divorce (Zhu and Qiao, 2015; Sun, 2018; Liu, 2019).

Thus, this study developed Hypotheses 5: When a wife's income exceeds that of her husband, a couple's marital satisfaction is decreased.

### Effects of Education on Marital Satisfaction

In addition, women who have more education and are employed are more resilient to marital disruptions and divorce petitions (Mincer, 1962; Bumpass et al., 1991; Nock, 2001). According to marriage gradient theory, men with higher education and income can improve couples' martial satisfaction when they hold traditional gender roles (Lu and Ruan, 2017). Evaluation of marital quality by wives is positively related to their husbands' education levels because husbands with higher education levels are more egalitarian in gender role attitudes (Vannoy and Philliber, 1992).

Thus, this study developed Hypotheses 6: When a wife is more educated than her husband, the couple's martial satisfaction is decreased.

### Effects of Women's Seniority on Marital Satisfaction

Few studies have investigated the effects of title and position. However, from the perspective of time allocation, it is assumed that when women have seniority in the occupational market, it will affect the time they allocate to home, leisure, and work activities, increasing family conflicts and indirectly influencing their spouse's marital satisfaction.

Thus, this study developed Hypotheses 7: When a wife holds a position of authority in the workplace, a husband's marital satisfaction is decreased.

### Effects of Family's Social Status on Marital Satisfaction

Female labor participation depends on the socioeconomic conditions of their family. Women from lower-income families are more likely to participate in the labor market and suffer more from work and family conflicts, which will ultimately affect marital satisfaction (Bumpass et al., 1991; Wu et al., 2015; Cai, 2019). When a husband's income satisfies household needs, women may choose to exit the labor market to parent at home (Wu, 2019).

Thus, this study developed Hypothesis 8: Married individuals from lower socioeconomic levels express lower marital satisfaction.

### Effects of Having a Child on Marital Satisfaction

Becker (1991) argues that there are mutual relationships among female labor participation rates, fertility rates, and female divorce rates; if couples are highly likely to divorce, they may have no children. Given the lack of childcare services in the market, society has reverted to the traditional roles of women as caregivers, leaving them with the ultimate responsibility (Ji, 2015; Qi and Dong, 2018). There is clearly a negative correlation in China between the number of underage children and their mothers' potential to participate in the labor market; it is more likely for mothers to exit the labor market when children are under the age of 6 (Ma et al., 2011; Jiang and Dai, 2019). In addition, Vannoy and Philliber (1992) argue that there are discrepancies in how the number of children and their ages affects couples' marital quality evaluations. Ye and Xu (2000) further argue in favor of a mutually connected relationship among a family life cycle, children, and marital duration, as children can decrease their parents' marital satisfaction. Mincer (1962) and Ogawa and Ermisch (1994) utilize the number of children at varying ages as a factor to test the variance of women's divorce risk.

Thus, this study developed Hypotheses 9: When wives are employed, an increase in the number of their children correlates with a decrease in couples' marital satisfaction.

### Effects of Kinship Support on Marital Satisfaction

Employed Chinese mothers in families of dual-earners may encounter work-to-family conflicts and tend to sacrifice their jobs for the family's sake. However, childcare responsibilities can be offset by kinship support customs, and a couple residing with elder relatives can lighten women's loads and positively mediate marital dissolution (Pimentel, 2000; Xiu and Gunderson, 2013; Song, 2019). Wu (2019) argues that employed women can also deal with domestic work division with elders in extended families. Indeed, Oishi and Oshio (2006) posit that there are no differences in the positive effects on female labor participation as a result of co-residing with either the husband or wife's parents. They also indicate the direct effects of co-residence on female labor participation, citing unclear effects on marital quality.

Thus, this study developed Hypotheses 10: For wives who are employed and co-reside with their parents aged below 70 years, there is a positive correlation with couples' marital satisfaction, but for wives who are employed and co-residing with parents aged above 70 years, there is a negative correlation.

## Materials and Methods

### Methods

In this analysis, a binary outcomes model was established:

(1) $$\begin{array}{r} Y=a+bX+e \end{array}$$

where *Y* is a binary dependent variable of marital satisfaction; *X* are the independent variables; *a* and *b* are coefficients; and *e* is the error term.

The distribution of dependent variables was uneven and skewed to a positive outcome, which defies the normal distribution prerequisite, and the logit model was used for binary choices. Robust cluster standard errors were applied to control the 26 different regions in the CFPS in 2014. A stability test was applied to further support female labor participation effects on marital satisfaction, and an adjusted dependent variable without a neutral response was chosen, revealing whether a neutral response affects the stability of the outcomes. Indeed, at this time, the distribution of the sample is extremely skewed.

### Data

The data used for analysis originated from the CFPS in 2014. China Family Panel Studies funded by Peking University and the National Natural Science Foundation of China, aim to collect and track data, including individuals, families, and regions, to reflect the transition of the Chinese society, economy, population, education, and health. CFPS began in 2010 and is maintained by the Institute of Social Science Survey of Peking University (Institute of Social Science Survey, 2014).

Whether there are gender differences in marital satisfaction is uncertain. Therefore, the data were first merged with the interviewee's spousal information. There were 33,591 couple-cases in the set, including 14,433 male interviewees and 14,538 female interviewees. Although some married couples' information has been displayed twice, with different interviewees, two cases were separately analyzed for each couple.

The aims of this analysis were to discuss the relationship between marital quality and female labor participation; hence, cases that were inconsistent with legal regulations of marriage and retirement were discarded. Based on Clause 1047 of the current Civil Law of the People's Republic of China, the legal marriage age for women is 20 years old. The statutory retirement age for women is uniquely differentiated among various firms; it is 55 years for women cadres and the self-employed, 50 years for female workers, and 60 years for senior female experts, while a majority of the women retire at 55 years. Thus, for the purpose of this study, the retirement age was considered to be 55 years. Retired female workers are included with those who are not in the labor market. Cases of cohabitating women were excluded, considering only participants joined in legal marriage.

Therefore, after adjustments by female age range and marital status, in sum, the dataset included 18,209 couple-cases, with 9,119 female interviewees and 9,090 male interviewees. In addition, each case includes information from interviewees and their spouses.

### Measures

#### Dependent Variable

##### Marital Satisfaction

Responses to the marital satisfaction question “*how satisfied are you in your marriage*” were gathered and ranked accordingly as 1 (no satisfaction), 2 (poor satisfaction), 3 (neutral), 4 (better satisfaction), and 5 (utmost satisfaction). Responses are skewed to better and utmost satisfaction. Subsequently, responses of no, poor, and neutral satisfaction were merged as negative marital quality evaluation, with an assigned value of null. Next, responses of better and utmost satisfaction were merged as positive marital quality evaluation, with an assigned value of one. Finally, the datasets were divided into two parts based on the gender of the interviewees. In the female dataset, the discussion focused primarily upon how wives' labor participation affects their marital satisfaction. Regarding the male dataset, the primary question considered how wives' labor participation affects husbands' marital satisfaction.

#### Alternative Dependent Variable for The Stability Test

##### Adjusted Marital Satisfaction

The variable of marital satisfaction was a binary category variable and included the neutral evaluation in the value of null. However, whether the interviewees were satisfied or dissatisfied with their marriage was difficult to present in the neutral evaluation. Therefore, neutral evaluation omitted to test whether holding neutral marital satisfaction affected stability of the outcomes. Adjusted marital satisfaction was still a binary variable ranging from 1 (no satisfaction) and 2 (poor satisfaction) to the value of null to present negative marital quality evaluation and from 4 (better satisfaction) and 5 (utmost satisfaction) to the value of one to present positive marital quality evaluation. However, the responses in the sample are extremely skewed to positive evaluation.

#### Independent Variables

##### Employment

This variable is a binary outcome describing the female occupation status. If the value of employment is one, wives are in the labor market. Otherwise, the value is null for those who are job seeking and who are not in the labor market. Stadelmann-Steffen (2011) argues that considering women's full-time employment and part-time employment is essential to assess their working condition and social situation. However, this study did not include part-time employment due to lack of applicable data.

##### Work Hours

According to a working time regulation of China's Labor Law, adopted in 1995, workers shall work for no more than 8 h a day and no more than 44 h a week. However, Qi and Dong (2018) note that overtime work is prevalent. Therefore, as a category variable, three levels of work time ranges were considered: no more than 40 h/week, between 40 and 60 h/week, and more than 60 h/week.

##### Types of Work

Demands, identity, and stress at work may lead to work and family conflicts and affect personal well-being (Mennino et al., 2005; Kossek et al., 2014; Moen et al., 2016). Not only the work itself but also the structure and order of the work affect physical and mental health. Occupation sometimes reflects cultural judgements of the ranking and importance of jobs (Eyles et al., 2018). Indeed, working women are a highly heterogeneous cohort and research should consider the distinctions in female occupational groups diversifying their marital evaluations. First, according to Xie et al. (2017), occupational codes changed from Chinese Standard Classification of Occupations (CSCO) to International Standard Classification of Occupation (ISCO-88). Through the process, some occupational codes were missed due to inappropriate match between CSCO and ISCO-88. Second, after the data check of samples in categories of the International Standard Classification of Occupations (International Labor Organization, 2004), the sample of Classification 1 legislators, senior officials, and managers and Classification 2 professionals merged into one category. The sample of Classification 6 craft and related trades workers and Classification 7 plant and machine operator and assemblers merged into one category, being at the same second ISCO skill level. Finally, types of female workers ranked as 0 (job seeking and not in the labor market), 1 (legislators, senior officials and managers, and professionals), 2 (technicians and associate professionals), 3 (clerks), 4 (service workers and shop and market sales workers), 5 (skilled agricultural and fishery workers), 6 (craft and related trades workers, and plant and machine operators and assemblers), and 7 (elementary occupations).

##### Relative Income

According to gradient marriage theory, women prefer to marry up socioeconomically (i.e., hypergamy). In addition, marital quality evaluation uses relative income value rather than absolute income value (Lu and Ruan, 2017). Angrist and Evans (1998) suggest that because some women are outside the labor market, it is difficult to rely only on women's wages for evaluation. Therefore, the subjective incomes of interviewees were evaluated to measure income effects. Responses to the question “*what is the level of your personal income in the city where you work*” were collected and rated with five rankings from the lowest to the highest. Subsequently, comparisons were made between interviewees' income statuses and those of their spouses. Finally, three levels of the categorical variable were utilized: couple income equivalent, husband income advantage, and wife income advantage.

##### Gender Role Attitudes

Responses were gathered from four gender attitude statements: “*A husband's job is to earn money; a wife's job is to look after the home and family*,” “*It is more important for a wife to help her husband's career than to pursue her own*,” “*Wives who have borne children have achieved their personal values*,” *and* “*Men ought to perform a share of household work*.” Responses were rated with five rankings: 1 (totally disagree), 2 (partially disagree), 3 (neutral), 4 (partially agree), and 5 (totally agree). The former three questions relate to traditional gender role attitudes. DeVellis (2011) argues that an alpha value of more than 0.7 denotes a good credential of scales, and a value between 0.60 and 0.70 is acceptable. The value of Cronbach's alpha for the former three questions was 0.6421, which is acceptable. However, if included, the fourth question largely decreases the credential levels. Therefore, the former three questions were used to evaluate the differences in gender role attitudes. Three measures were added to eliminate the dimension by standardizing extreme values. With a baseline value of 0.5, if the standardized values are close to 1, the interviewee's gender role attitude tends to be traditional; otherwise, if close to 0, it tends to be more modern. Interviewees and their spouses were assigned to four categories, including both traditional and modern gender attitudes, or it was recognized if the couple held opposite gender role attitudes. Finally, an intersection variable with gender role attitudes and female labor participation was established, which evaluated how interviewees and their spouses' gender role attitudes affect couples' marital satisfaction separately when the wife is in the labor market.

##### Husband's Domestic Work Time

The variable from the dataset regarding how husbands spend time on domestic work was selected. Three categories on how the husband's time was spent were used: no participation in domestic work; 1 h or less a day; and more than 1 h a day. These three categories were nearly evenly distributed.

##### Education

The variable of highest educational qualification has nine educational levels. Considering the different weighting of each level, the study combined illiteracy, no need of schooling, and level of elementary school into less than elementary school level. Further, levels of college, bachelor, master, and Ph.D. belonged to beyond high school levels. Finally, the variable of highest educational qualification was arranged into three categories: less than elementary school level, at middle school, and beyond high school levels. These three categories were equivalent in weights, and each represented nearly one-third of the distribution. Comparisons among couples were then made to develop three categories: couple's education equivalent, husband's education advantage, and wife's education advantage.

##### Age

Couples' age differences affect marital stability and women's divorce risks (Ogawa and Ermisch, 1994; Chen and Qin, 2019). With the traditional age-difference preferences in the marriage market in China, it is socially acceptable to have marital patterns of older husbands with younger wives or minor age differences between elder wives and younger husbands (Chen and Qin, 2019). In this analysis, wives' ages were subtracted from husbands' ages to develop five age-difference-ranges: couple equivalent in age, wives older or younger than husbands within 5-year age differences, and wives older or younger than husbands beyond 5-year age differences.

##### Child

Previous research has supported that the number of children affects female labor participation and marital quality. In the analysis, the number of children is a category variable from zero to nine children at home. Considering the effects of preschool children, a binary variable was utilized to test whether children under the age of six are living at home. If the response was yes, the value of one was assigned and null otherwise. Finally, there were two intersectional variables of female employment status: number of children with female labor participation status, and existence of preschool children with female labor participation status. These were tested separately to determine how female labor participation under children effect affects couples' marital satisfaction.

##### Position of Authority

Position of authority is a binary variable; one was assigned for women achieving title/position and null otherwise.

##### Co-residence With Elderly Parents

Song (2019) posits that residing with elders is beneficial to young married wives' labor participation, but the situation changes adversely when the elderly require home support. The analysis presented two binary category variables to clearly show the effects of parents' age. If the younger couples resided with the husbands' or wives' parents aged under 70 years, the binary variable was a positive value; otherwise, it was null. If the younger couples resided with their parents aged over 70 years, the binary variable was one; otherwise, it was null. Female labor participation and co-residence with parents aged either under or over 70 years were intersected to test the effects on marital satisfaction.

##### Family Social Status

Responses were solicited to the question “*how do you measure your family social status in the city where you work*” with rankings from 1 (lowest), 2 (lower), 3 (medium), and 4 (higher) to 5 (highest). Rankings of 1 and 2 were combined as low social status and 4 and 5 combined as high social status. Finally, the variable of family social status has three levels: low, medium, and high social status.

##### Urban

Based on the dualistic structure system of urban and rural areas, there are clear economic, female labor participation, and marital quality differences within urban and rural areas (Xu, 2010). Thus, urban is a binary variable when interviewees live in an urban area are assigned a positive value; otherwise, the value is null.

## Results and Conclusions

Table 1 shows a statistical summary of all variables. To discuss gender differences in marital satisfaction, two sample sets were arranged by gender. The chi-square test outcomes show that marital satisfaction has gender differences. A total of 83.9% of women and 90.3% of men experienced positive marital satisfaction, revealing a skewed distribution of marital satisfaction. After adjustment of marital satisfaction, the distribution of the sample was skewed extremely to positive marital satisfaction. 78.49% of women were in the labor market. Half of women worked in the service and agriculture industries. Nearly 60% of women were not in the labor market or worked no more than 40 h/week, within the labor law work-time regulations. Nearly half the cases were in equal situations in terms of income status, and only a quarter of the cases had women in a higher income status than their counterparts. Regarding gender role attitudes, more than 70% of the women and men held traditional gender roles, with more than 70% of the men sharing domestic work responsibilities at home. Half of the couples shared identical educational levels. In nearly 50% of the couples, the husband was older than the wife with a 5-year age difference. The marriage match patterns of education and age were consistent with social conventions. The average number of children at home consisted of no more than two, and nearly half of the cases had only one child. Considering mothers' working conditions, the average number of children had little effect, which partly supports the negative relationship between female labor participation and fertility; however, there is still little evidence of causality. Few women in the labor market had titles or positions, with half of the cases at medium social levels. About one-fifth of cases co-resided with parents aged below 70 years when the woman was employed; otherwise, only 10% of the couples co-resided with parents aged above 70 years. Couples were distributed equally among urban and rural regions. In sum, women's labor participation and couples' marital satisfaction were independent, even in the adjusted marital satisfaction with a precise level of significance.

**Table 1:** Descriptive statistics of the variables.

**Gender of interviewees**	**Female**				**Male**			
**Measures**	**Obs**.	**Mean**	**Se**	**Range**	**Obs**.	**Mean**	**Se**	**Range**
Marital satisfaction^***^	8,189	0.839	0.368	[0,1]	7,868	0.903	0.297	[0,1]
Adjusted marital satisfaction^***^	7,217	0.952	0.214	[0,1]	7,326	0.969	0.173	[0,1]
Employment	8,946	0.784	0.411	[0,1]	8,914	0.785	0.411	[0,1]
Types of employment								
Job seeking and not in the labor market	8,179		(base)		8,146		(base)	
Legislators, senior officials and managers, and Professionals	8,179	0.053	0.224	[0,1]	8,146	0.052	0.223	[0,1]
Technicians and associate professionals	8,179	0.025	0.156	[0,1]	8,146	0.025	0.157	[0,1]
Clerks	8,179	0.032	0.175	[0,1]	8,146	0.031	0.174	[0,1]
Service workers and shop and market sales workers	8,179	0.138	0.345	[0,1]	8,146	0.138	0.345	[0,1]
Skilled agricultural and fishery workers	8,179	0.392	0.488	[0,1]	8,146	0.392	0.488	[0,1]
Craft and related trades workers and Plant and machine operators and assemblers	8,179	0.087	0.282	[0,1]	8,146	0.088	0.283	[0,1]
Elementary occupations	8,179	0.038	0.191	[0,1]	8,146	0.038	0.192	[0,1]
Work no more than 40 h	9,119		(base)		9,090		(base)	
Work between 40 and 60 h	9,119	0.258	0.438	[0,1]	9,090	0.258	0.438	[0,1]
Work more than 60 h	9,119	0.144	0.351	[0,1]	9,090	0.144	0.351	[0,1]
Equivalent income couple	7,826		(base)		7,809		(base)	
Husband's income advantage	7,826	0.318	0.466	[0,1]	7,809	0.318	0.466	[0,1]
Wife's income advantage	7,826	0.225	0.418	[0,1]	7,809	0.225	0.417	[0,1]
Female gender role attitudes	8,167	0.741	0.228	[0,1]	8,147	0.742	0.227	[0,1]
Male gender role attitudes	7,855	0.711	0.232	[0,1]	7,842	0.711	0.232	[0,1]
Husband's domestic work time per day								
No participation	8,536		(base)		8,507		(base)	
Less than 1 h	8,536	0.403	0.491	[0,1]	8,507	0.404	0.491	[0,1]
More than 1 h	8,536	0.313	0.464	[0,1]	8,507	0.312	0.463	[0,1]
Equivalent education couple	9,119		(base)		9,090		(base)	
Husband's education advantage	9,119	0.282	0.450	[0,1]	9,090	0.282	0.450	[0,1]
Wife's education advantage	9,119	0.145	0.352	[0,1]	9,090	0.144	0.351	[0,1]
Equivalent age couple	9,119		(base)		9,090		(base)	
Wife older by 5 years or less	9,119	0.172	0.377	[0,1]	9,090	0.173	0.378	[0,1]
Husband older by 5 years or less	9,119	0.546	0.498	[0,1]	9,090	0.546	0.498	[0,1]
Wife older by more than 5 years	9,119	0.004	0.067	[0,1]	9,090	0.005	0.068	[0,1]
Husband older by more than 5 years	9,119	0.112	0.315	[0,1]	9,090	0.111	0.315	[0,1]
Number of children	9,119	1.503	0.889	[0,9]	9,090	1.510	0.890	[0,9]
Number of children with employed mother	8,946	1.212	1.017	[0,9]	8,914	1.214	1.018	[0,9]
Preschool children with employed mother	8,946	0.202	0.401	[0,1]	8,914	0.203	0.402	[0,1]
Position of authority	9,119	0.025	0.156	[0,1]	9,090	0.025	0.156	[0,1]
Medium family social status	8,173		(base)		7,858		(base)	
Low family social status^**^	8,173	0.191	0.393	[0,1]	7,858	0.184	0.388	[0,1]
High family social status^***^	8,173	0.287	0.452	[0,1]	7,858	0.266	0.442	[0,1]
Co-residence of 70 or less with employed female	8,946	0.237	0.425	[0,1]	8,914	0.236	0.425	[0,1]
Co-residence of more than 70 with employed female	8,946	0.098	0.297	[0,1]	8,914	0.098	0.298	[0,1]
Urban	8,615	0.473	0.499	[0,1]	8,393	0.482	0.500	[0,1]
**Chi-square test**								
**Variables**			**Pearson** **χ^2^**		**d.f**.		***p*****-value**	
Marital satisfaction and Employ			0.517		1		0.472	
Adjusted marital satisfaction and Employ			3.710		1		0.054	

*Variables with gender differences were checked by the chi-square test and marked with*

*** *p < 0.01*,

** *p < 0.05*,

* *p < 0.10*.

Table 2 illustrates how female labor participation produces insignificant effects on marital satisfaction. There was an insignificant 16% negative impact of female labor participation on men's marital satisfaction. Women who worked more than 60 h a week experienced a 24% decrease in their marital satisfaction, compared to women who were not in the labor market or worked <40 h. Women insignificantly increased their marital satisfaction within the range of 40–60 h/week. However, men's marital satisfaction increased insignificantly when women worked more than 60 h.

**Table 2:** Estimated logistic regression coefficients from female occupation status (Model 1) and stability test (Model 2) predicting marital satisfaction.

	**Model 1** **Marital satisfaction**		**Model 2** **Adjusted marital satisfaction**	
**Gender of interviewees Variables**	**Female**	**Male**	**Female**	**Male**
Employment^a^	0.026 (0.119)	−0.173 (0.166)	−0.322 (0.198)	−0.201 (0.256)
Work between 40 and 60 h	0.065 (0.096)	−0.014 (0.091)	0.297^*^ (0.157)	0.082 (0.176)
Work more than 60 h	−0.264^***^ (0.133)	0.094 (0.077)	−0.051 (0.196)	0.473^***^ (0.235)
Husband's income advantage	−0.156 (0.108)	0.003 (0.087)	−0.531^***^ (0.134)	0.092 (0.215)
Wife's income advantage	−0.169^*^ (0.093)	−0.259^***^ (0.096)	−0.164 (0.130)	−0.280 (0.196)
Gender role attitudes when female employed^b^				
Both egalitarian	0.689^***^ (0.208)	0.270 (0.278)	0.194 (0.419)	0.596 (0.591)
Interviewee egalitarian but spouse traditional	0.165 (0.142)	0.110 (0.160)	0.318 (0.272)	−0.029 (0.286)
Interviewee traditional but spouse egalitarian	0.080 (0.149)	0.352 (0.284)	0.105 (0.231)	0.102 (0.381)
Both traditional	0.196^***^ (0.077)	0.309^***^ (0.145)	0.291(0.228)	0.356 (0.221)
Husband's domestic work time per day				
Less than 1 h per day	0.105 (0.078)	0.053 (0.124)	0.048 (0.195)	−0.151 (0.183)
More than 1 h per day	0.118 (0.121)	−0.096 (0.183)	0.180 (0.211)	−0.143 (0.141)
Husband's education advantage	−0.091 (0.074)	−0.163^*^ (0.094)	−0.014 (0.137)	−0.155 (0.209)
Wife's education advantage	−0.167 (0.107)	−0.014 (0.121)	−0.071 (0.168)	−0.210 (0.223)
Wife older by 5 years or less	0.227^***^ (0.085)	0.061 (0.167)	0.304^***^ (0.154)	0.326 (0.324)
Husband older by 5 years or less	−0.035 (0.097)	−0.101 (0.137)	−0.327^*^ (0.190)	−0.129 (0.215)
Wife older by more than 5 years	0.978 (0.678)	−0.474 (0.619)	0.289 (0.869)	−1.177 (0.759)
Husband older by more than 5 years	−0.205^*^ (0.119)	−0.198 (0.181)	−0.338 (0.224)	−0.057 (0.221)
Children with employed mother	−0.131^***^ (0.054)	−0.127^*^ (0.067)	−0.134 (0.087)	−0.286^***^ (0.114)
Preschool children with employed mother	0.166 (0.102)	−0.011 (0.101)	0.403^***^ (0.157)	0.099 (0.195)
Position of authority	0.259 (0.301)	0.165 (0.254)	0.660 (0.775)	0.722 (0.660)
Low family social status	−0.592^***^ (0.095)	−0.454^***^ (0.144)	−0.907^***^ (0.182)	−0.910^***^ (0.291)
High family social status	0.597^***^ (0.121)	0.338^***^ (0.147)	0.068 (0.132)	−0.259 (0.218)
Co-residence of parents aged under 70 with employed female	0.050 (0.088)	0.079 (0.075)	0.175 (0.165)	−0.010 (0.114)
Co-residence of parents aged over 70 with employed female	−0.080 (0.111)	−0.016 (0.092)	−0.028 (0.147)	0.047 (0.194)
Urban	0.320^***^ (0.075)	0.224^***^ (0.101)	0.561^***^ (0.157)	0.362^*^ (0.203)
Constant	1.584^***^ (0.150)	2.474^***^ (0.191)	3.405^***^ (0.272)	4.157^***^ (0.379)
Observations	7,020	6,981	6,202	6,516

*** *p < 0.01*,

** *p < 0.05*,

* *p < 0.10. Clustered robust standard errors are in parentheses*.

a *Reference categories are sequential: women are not in the labor market or job seeking; work no more than 40 h; equivalent income couple; husband with no participation in domestic work; equivalent education couple; equivalent age couple; medium family social status and living in rural area*.

b *Both egalitarian = interviewee and her/his spouse held same egalitarian gender attitude when female employed; Interviewee egalitarian but spouse traditional = interviewee held egalitarian while her/his spouse held traditional gender attitude when female employed; Interviewee traditional but spouse egalitarian = interviewee held traditional while her/his spouse held egalitarian gender attitude when female employed; Both traditional = interviewee and her/his spouse held same traditional gender attitude when female employed*.

When women are in the labor market, couples who hold the same gender attitudes increase their marital satisfaction at a high rate. Indeed, women with modern attitudes increased their marital satisfaction close to 78% more significantly than women with traditional gender attitudes. In addition, men showed positive marital satisfaction with female labor participation regardless of gender attitudes. Only if men are performing domestic chores will female marital satisfaction increase insignificantly. Marital satisfaction of men who devoted time to housework beyond 1 h a day insignificantly decreased by 10%.

Compared to equivalent income statuses, marital satisfaction decreased by about 16% for women and 23% for men under the condition that income statuses of women were higher than those of their spouses. Educational differences among couples had negative effects on the marital satisfaction of husbands and wives, especially for men with higher education than their wives. Age matches were diversified with marital satisfaction. Analyses of men were insignificant in terms of age differences. However, women who were older than their spouses by 5 years or less experienced 25% greater marital satisfaction than age equivalent couples.

The marital satisfaction of couples was sensitive to the number of children in the marriage. Couples' marital satisfaction decreased by nearly 12% separately for each additional child if women were in the labor market. However, if there were preschool-aged children, women's marital satisfaction increased but there were no differences between spouses. Whether couples had positions of authority had positive but insignificant effects on marital satisfaction. Couples with lower status were more dissatisfied with their marriage. Co-residence with parents for working women had conversely insignificant effects on marital satisfaction, which depended on parents' age. Couples living in urban regions had a nearly 30% higher marital satisfaction value.

In Model 2, an adjusted dependent variable was chosen to test the stability of the findings. With omitted neutral responses of marital satisfaction, the distribution of sample was extremely skewed to positive values. Women's labor participation still had no statistical significance for couples' marital satisfaction. Women's response to workloads registered similarly as they are sensitive to working between 40 and 60 h, gaining an increase in 34% marital satisfaction; and working more than 60 h/week, suffering a decrease in 5% marital satisfaction. Men experienced a significantly higher marital satisfaction of 60% when women worked more than 60 h/week, compared to when women worked <40 h or were not in the labor market.

Effects on marital satisfaction are still positive if couples hold the same gender attitudes. In a narrowed sample, unemployed women were compared with couples who both hold modern gender role attitudes; women's marital satisfaction increased by 21% and men's marital satisfaction increased by nearly 80%. Men sharing domestic work still accelerates women's marital satisfaction but decreases men's satisfaction when they work more than 1 h a day.

Matching relative income still affects marital satisfaction. Couples had lower marital satisfaction when wives' income status exceeded that of their husbands, with a 25% reduction for men. Disparities in educational levels shrank marital satisfaction. Marital satisfaction dramatically decreased when men were <5 years older than their female counterparts.

When women were in the labor market, the presence of preschool children increased wives' marital satisfaction. With each additional child, couples' marital satisfaction decreased by 25% for men. In terms of low social status, seniority, and co-residence with parents were mostly the same as in Model 1.

Different occupations have implicitly required different working time input and work styles, and have caused subsequent differences in income and social status. In Table 3, when other related variables are controlled, some occupational groups had specific differences on marital satisfaction. Women working as legislators, senior officials, managers, and professionals had significant positive effects on couples' marital satisfaction, with a rise of 62% for women. Women working as clerks have significant negative effects on husbands' marital satisfaction, with a decrease of 40% for men. Women working in services and agriculture, presented in the stability model, decreased their own marital satisfaction nearly 40%. Women's work hours still had same effects on their marital satisfaction, with positive responses between 40 and 60 h but negative responses beyond 60 h.

**Table 3:** Estimated logistic regression coefficients from female occupation groups (Model 1) and stability test (Model 2) predicting marital satisfaction.

	**Model 1** **Marital satisfaction**		**Model 2** **Adjusted marital satisfaction**	
**Gender of interviewees Variables**	**Female**	**Male**	**Female**	**Male**
Types of employment^a^				
Legislators, senior officials and managers, and professionals	0.485^**^ (0.231)	0.345 (0.228)	0.334 (0.506)	0.538 (0.543)
Technicians and associate professionals	0.310 (0.199)	−0.047 (0.298)	1.447 (0.942)	0.551 (1.021)
Clerks	0.049 (0.202)	−0.477^*^ (0.263)	−0.513 (0.333)	−0.627 (0.488)
Service workers and shop and market sales workers	0.016 (0.123)	−0.017 (0.190)	−0.501^*^ (0.257)	−0.009 (0.387)
Skilled agricultural and fishery workers	0.039 (0.122)	−0.271 (0.192)	−0.474^**^ (0.213)	−0.355 (0.296)
Craft and related trades workers and plant and machine operators and assemblers	−0.128 (0.146)	−0.158 (0.234)	−0.261 (0.245)	−0.053 (0.351)
Elementary occupations	0.285 (0.193)	−0.139 (0.289)	−0.165 (0.324)	−0.288 (0.334)
Work between 40 and 60 h	0.101 (0.100)	−0.016 (0.105)	0.288^*^ (0.169)	0.074 (0.171)
Work more than 60 h	−0.207^*^ (0.118)	0.089 (0.096)	−0.067 (0.199)	0.349 (0.264)
Other variables controlled	Yes	Yes	Yes	Yes
Constant	1.626^***^ (0.150)	2.422^***^ (0.202)	3.411^***^ (0.270)	4.155^***^ (0.384)
Observations	6,658	6,615	5,890	6,179

*** *p < 0.01*,

** *p < 0.05*,

* *p < 0.10. Clustered robust standard errors are in parentheses*.

a *Reference category is that women are not in the labor market or job seeking*.

The analysis determines that whether wives participate in the labor market has no effect on their own or their counterparts' marital satisfaction, which contradicts Hypothesis 1. This conclusion follows Vannoy and Philliber (1992), Blair (1998), and Helms et al. (2018), who declare that wives' employment has limited effect on couples' marital satisfaction. Wives who work more than 60 h/week negatively affect their marital satisfaction, which contradicts Hypothesis 4 in terms of workload. This conclusion follows Greenstein (1990) and Amato et al. (2003) who argue that women's extended hours of employment lead to a decline in their marital quality.

The findings in gender role attitude effects are beyond expectations, contradicting Hypothesis 2. First, regardless of the held gender roles, women who are in the labor market have increased couples' marital satisfaction, regardless of insignificant coefficients. Second, couples with the same gender role attitudes have higher marital satisfaction than those with different gender attitudes when women's employment conditions are compared. Indeed, couples with the same modern gender role attitudes rate their marital satisfaction significantly higher than holders of traditional attitudes. Third, women who hold traditional gender role attitudes but are employed have the smallest increase in marital satisfaction.

Husbands sharing domestic work at home have insignificant but positive effects on women's marital satisfaction. However, men's longer hours of devotion to housework decreases their marital satisfaction, which partially supports Hypothesis 3.

Hypothesis 5 is supported by the findings; wives' income exceeding that of their husbands will significantly decrease couples' marital satisfaction. Age and education matching is still crucial to marital satisfaction. Hypothesis 6 is fully supported–disparities in educational levels reduce not only wives' but also husbands' marital satisfaction. This conclusion is consistent with the argument by Vannoy and Philliber (1992) that wives' evaluation of marital quality is positively related to husbands' educational background.

When women are in the labor market, the effects of positions of authority and co-residing with their parents regardless of parents' age are statistically insignificant, which contradicts Hypotheses 7 and 10. Children, as an important factor, have the same effects as found in previous studies, in which one additional child at home significantly decreases couples' marital satisfaction, supporting Hypothesis 9. Regarding Hypothesis 8, lower social status of the family leads to lower marital satisfaction for the couple.

## Discussion

Employment effects on marital satisfaction are more significantly related to time scheduling between work and family rather than whether women are in the labor market. As salary earners, Chinese women's dual roles have been widely accepted, demonstrated by institutional support from the period of the planned economy and women's independent labor participation to that of the market economy (Shu and Zhu, 2012). Furthermore, parental education is an indirect factor that influences the interviewees' attitude that women's work, educational levels, and mothers' occupation status are beneficial to children's socialization and transmission of values (Kosteas, 2013). Therefore, the culture of mothers' participation in the labor market has promoted social acceptance of women's work.

However, employed women increasingly encounter work and family conflicts, which may affect couples' marital satisfaction. About 70% of workers have reported work and family conflicts in the US, and many employed women struggle to make the integration between work responsibilities and family needs (Minnotte et al., 2013; Kossek et al., 2014). Since 2000, women in China who experience work and family conflicts increasingly tend to leave the labor market (Zhu, 2011). China Family Panel Studies data from 2014 indicate that in 2,664 of 3,842 cases, 69% of the women were not in the labor market owing to caregiving issues, including fertility, childcare, and domestic work.

Work and family conflicts arise from inequality of social recognition of market work and domestic work, and gender inequality. In public sphere, society regards that the value of market work outweighs the value of domestic work. Indeed, work institutions regard rewarding employees as those who are full-time committed to the jobs without family obligation-related breaks (Mennino et al., 2005; Kossek et al., 2014). Gender inequality implies that women and men unequally share not only house chores and childrearing in domestic work but also responsibilities and rewards in market work (Mennino et al., 2005). Indeed, Ma and Rizzi (2017) argue that while egalitarian attitudes have been fully accepted by women, they are reluctantly adopted by men. Wives who have egalitarian attitudes but deal with unequal domestic work divisions are more likely to report low marital satisfaction (Wilcox and Nock, 2006). Furthermore, Mennino et al. (2005) find negative effects on individual's behaviors and moods when the needs of work and family are competing for personal time, energy, and attention. Cao (2019) also delineates characteristics of work and family conflict as time conflict, role conflict, and pressure conflict. Role conflict is due to women's dual or even triple roles as employees, wives, and caregivers, and is often connected with gender role attitudes.

Role, time, and pressure conflicts are interconnected. Pressure conflict originates from the role of being a good mother and the financial support of intensive childcare. Hays (1996) mentioned intensive mothering and defined good parenting as “child-centered, expert-guided, emotionally absorbing, labor intensive and financially expensive.” (Hays, 1996, p. 8). Budds (2021) found that intensive mothering as a normative standard had been identified among countries like the UK, the US, Australia, and Sweden. Elliott et al. (2013) argue that intensive mothering led to the rise of mothers' pressure, regardless of different racial/ ethnic and social-economic mother cohorts. Mothers can easily blame themselves for the problems their children encounter. Intensive mothering is common in China. In addition, there is a tendency to criticize mothers prioritizing work over motherhood. Cui (2017) argues that ambitious women are socially accepted in the workplace but are regarded as irresponsible in performing family duties at home. Almost 70% of women are employed, and many women experience role conflict between being a good mother and an employee (Zheng, 2019). With the mutual effects of the one-child policy and the development of market economy, the fertility rate and the number of children decreased, and simultaneously, accumulated resources have centered on investment in children, which finally brings about child supremacy (Zheng, 2019). Based on the China Family Panel Study in 2014, Ma (2018) estimated that direct care costs for a child from birth to 17 years of age are 191,000 yuan on average, 273,200 yuan in urban areas, and 143,400 yuan in rural areas. Indeed, the cost of raising children in low-income families is significant. Hui (2017) further analyzed direct economic costs from preschool-age children, showing that the annual average cost is 6,561 yuan, 10,297 yuan in urban areas, and 5,945 yuan in rural areas. Therefore, female labor participation is required to support the extensive costs of caring for children. Consequently, time conflict reveals that married couples have allocated most of their time to work and children, leaving a shortage of time to devote to themselves and each other.

Previous research has mentioned effects of part-time work, marital duration, and types of marriage on marital satisfaction. Due to insufficient applicable samples, this study did not cover them. In addition, the dependent variable in the analysis is marital satisfaction, which is a one-way measure of marital quality. With the limitation of the dataset, marital satisfaction is the only subjective evaluation of marriage quality. Therefore, acquiring access to more characteristics of marital quality could further provide more intensive research on effects.

However, evaluations of marriage quality cannot directly determine couples' marital stability. In the consecutive research of CFPS in 2016, no more than 20 cases resulted in divorce, which reveals that marriages with low marital satisfaction can endure. Xu (2012) further argues that although some interviewees admitted that their marriage could easily dissolve, marriage in China is still highly cohesive and stable. Jiang and Dai (2019) further argue that creating a much more flexible work form is an important policy to promote female labor participation. Further research is required in the context of COVID-19 and to investigate the effects on marital quality when more women choose to work from home. As Platte (1988) argued, every time a new revision to the Marriage Law is enacted in China, it promotes a sudden increase in the divorce rate. After the enforcement of the Civil Law, changes in marital dissolution and how the new law aids individuals to meet the requirements of divorce should be researched.

## Data Availability Statement

Publicly available datasets were analyzed in this study. This data can be found here: China Family Panel Studies, Peking University Open Research Data at https://doi.org/10.18170/DVN/45LCSO.

## Author Contributions

XY and SL: conceptualization and methodology. SL: writing—original draft preparation. XY: writing—review and editing. Both authors have read and agreed to the published version of the manuscript.

## Conflict of Interest

The authors declare that the research was conducted in the absence of any commercial or financial relationships that could be construed as a potential conflict of interest.
